# Analytical and clinical performance of the fully-automated LIAISONXL calprotectin immunoassay from DiaSorin in IBD patients

**DOI:** 10.1016/j.plabm.2020.e00175

**Published:** 2020-06-24

**Authors:** R. Vicente-Steijn, J.M. Jansen, R. Bisheshar, I.-A. Haagen

**Affiliations:** a Laboratory of Hematology and Clinical Chemistry, OLVG Oost, Amsterdam, the Netherlands; b Department of Gastroenterology and Hepatology, OLVG Oost, Amsterdam, the Netherlands

**Keywords:** Intestinal inflammation, Fecal calprotectin, Ulcerative colitis, Crohn’s disease

## Abstract

**Objectives:**

Distinction between inflammatory bowel disease (IBD) and irritable bowel syndrome (IBS) based on clinical symptoms is often difficult. In this study we assessed the performance of the fully-automated calprotectin immunoassay from DiaSorin in IBD diagnosis and follow-up and compared it to the EliA calprotectin 2 immunoassay.

**Design:**

and Methods: The calprotectin immunoassay from DiaSorin run on the LIAISONXL was analytically and clinically validated and compared to the EliA calprotectin 2 immunoassay from Thermo Fisher Scientific run on the ImmunoCAP250. Five patient groups were measured (n ​= ​303): IBD: ulcerative colitis (UC) and Crohn’s disease (CD); non-IBD: IBS, other gastrointestinal diseases and controls (healthy patients with no gastrointestinal disease).

**Results:**

The calprotectin immunoassay of DiaSorin showed good analytical performance with frozen samples. The presence of blood in the stool can interfere with the measurement of calprotectin. Patients suffering from IBD (UC or CD) showed significant higher concentrations of fecal calprotectin compared to controls (UC:710 ​± ​921 ​mg/kg; CD:967 ​± ​1243 ​mg/kg; controls:11±8 ​mg/kg) using DiaSorin’s immunoassay. The remaining non-IBD groups showed no significant difference compared to controls. Follow-up patients (n ​= ​9) showed a significant decrease in fecal calprotectin after treatment. At 50 ​mg/kg cut-off value, the negative predictive value for DiaSorin’s immunoassay was 96% and the positive predictive value 83% (sensitivity of 95% and specificity of 86%).

**Conclusions:**

The lack of standardization contributes to the numerical differences between the two methods, but the qualitative conclusions do not differ. DiaSorin’s calprotectin immunoassay can be used both to distinguish between IBD and non-IBD patients as well as for follow-up of IBD patients.

## Highlights

• New calprotectin immunoassay has a short sample preparation time and is easy to use.
• DiaSorin’s immunoassay can be used to distinguish between high and low risk IBD patients, as opposed to IBS.
• Differences observed between immunoassays are due to lack of standardization.
• Blood in the stool can effect the calprotectin measurement.

## 1 Introduction

In the last decades, incidence of inflammatory bowel disease (IBD) is increasing [1]. This chronic intestinal inflammation disorder comprises two major forms of inflammation: Crohn’s disease (CD) and ulcerative colitis (UC). Still 5–10% of patients suffer from unclassified IBD, where no definitive diagnosis is possible. Although both CD and UC have distinct pathologic and clinical characteristics, their pathogenesis remains poorly understood [2,3].

Symptoms that lead to a first examination with suspicion of IBD include persistent (longer than 4 weeks) or recurrent (more than 2 episodes in the past 6 months) abdominal pain and diarrhoea. Additional symptoms like rectal bleeding, weight loss or anaemia increase the chances of diagnosing IBD [3]. The golden standard to ascertain a diagnosis is through endoscopic evaluation and histological sampling, which is frequently considered uncomfortable by patients [3,4]. When diagnosing IBD, a relative high amount of adults will show no endoscopic abnormalities although other symptoms might be present [5]. Alternatively, chronic abdominal pain and altered bowel movements can also be the result of irritable bowel syndrome (IBS). It is the most commonly diagnosed gastrointestinal condition lacking an organic cause [6]. Thus, identifying low risk patients is imperative to avoid unnecessary invasive procedures. On the other hand, the need to identify patients with a high likelihood of IBD is high to justify urgent colonoscopy. Another important aspect of managing a disease like IBD is monitoring the extent and severity of the inflammation. In case new symptoms develop, assessing whether these reflect a change in the inflammatory activity is crucial to optimize therapy only when required and avoid unnecessary endoscopic evaluation when possible [7,8].

Multiple studies have proven the use of calprotectin in IBD patients as a biomarker in monitoring disease activity, response to treatment and relapse [[7], [8], [9], [10], [11]]. Calprotectin levels directly correlate with the amount of granulocytes present at the intestinal inflammation site, correlating with the severity of IBD [[12], [13], [14]]. Because of its booming success, different assays to determine fecal concentration of calprotectin have arisen in a relative short period of time [[13], [14], [15], [16], [17], [18]]. The amount of patients being screened with calprotectin for IBD is increasing to avoid unnecessary endoscopy, thus a need for an easy to use and quick laboratory assay arises. Not only that, but the measurement of calprotectin concentration in monitoring IBD patients is practiced more often [7,8]. Currently, three main technologies are being used: enzyme immunoassay (ELISA/EIA), chemiluminescent immunoassay (CLIA) and turbidimetry. Studies are necessary to establish whether the different assays show comparable clinical sensitivities and specificities and share the same cut-off values.

In this study we evaluate the recently developed calprotectin immunoassay from DiaSorin for the LIAISON® XL (CLIA). This novel CLIA assay from DiaSorin uses as solid phase paramagnetic particles coated with a mouse monoclonal antibody against calprotectin and a second, conjugated mouse monoclonal antibody against a different region of calprotectin. The novelty in DiaSorin’s CLIA assay are the calibrators and controls using ultra pure recombinant human calprotectin dimers. These dimers were produced, purified and standardized to MS/MS because of the lack of standardization among commercially available calprotectin assays. In this study we compare the DiaSorin CLIA assay to the widely used EliA calprotectin 2 immunoassay (EIA) for the ImmunoCAP250 developed by Thermo Fisher Scientific (TFS) and determine whether it is suitable to distinguish between high and low risk IBD patients (to avoid unnecessary endoscopy) and monitor patients suffering from IBD.

## 2 Materials and methods

### 2.1 Patients

An overview of the patients included in this study is shown in Table 1. A total of 5 patient groups were studied: CD, UC,IBS, other gastro-intestinal (GI) diseases and controls. The calprotectin immunoassay from DiaSorin was evaluated on a total of 303 patients from the OLVG Oost Hospital in Amsterdam consulted by a gastroenterologist. Diagnosis of IBD (CD or UC) was confirmed by endoscopy followed by histology according to the Dutch IBD Guideline for adults [2,3,6]. Only patients diagnosed with either CD or UC without any medication at the time of diagnosis were included in this analysis. This study included two types of IBD patients: the first type are patients diagnosed *de novo* following the Dutch IBD guideline as previously mentioned and showing two independent elevated calprotectin measurements; the second type are previously diagnosed IBD patients with a new exacerbation episode after a prolonged period (>1 year) without IBD-related medication. Patients with unclassified IBD were not included in this study. IBS patients were diagnosed according to symptoms upon presentation [6]. An additional group of patients presenting with other gastro-intestinal (GI) diseases was also included (suffering of for e.g. unexplained diarrhoea, bacterial inflammation or gastritis). The control group comprised a group of patients screened for IBD but without any intestinal symptoms or a history of intestinal disease. The samples used in this study were obtained from patients who provided no objection to the use of their samples for research purposes. The study was approved by the hospital’s ethical committee.

**Table 1:** Overview of patients included in the study. CD: Crohn’s disease; UC: ulcerative colitis; IBS: irritable bowel syndrome; Other GI: other gasto-intestinal disease.

Group	CD	UC	IBS	Other GI	Control
Number	54	73	82	53	41
Median age (interquartile range)	37 (25)	47 (30)	36 (17)	43 (28)	39 (22)
Male:Female (n)	19:35	36:37	23:59	20:33	16:25
[fecal Calprotectin] Diasorin ​± ​SD mg/kg	967 ​± ​1243	710 ​± ​921	23 ​± ​43	53 ​± ​68	11 ​± ​8
[fecal Calprotectin] Thermo Fisher ​± ​SD mg/kg	1294 ​± ​1255	1041 ​± ​1090	27 ​± ​39	96 ​± ​279	12 ​± ​7

### 2.2 Sample collection, preparation and extraction

The patients provided fecal samples collected at home using a standard sample container provided by the laboratory. The samples were collected in multiple laboratory locations were a secondary sample was collected in a universal spoon tube. Upon arrival at the clinical laboratory samples were taken and immediately frozen (−20 ​°C) or were sent in frozen state and analysed in batches. The samples were stored frozen up to 1 week before measuring calprotectin concentrations. Upon thawing, extracts of stool samples were simultaneously prepared using both kits to measure calprotectin levels with both immunoassays in the same day (Liaison and ImmunoCAP250).

The Calprotectin Stool Extraction Device (DiaSorin S.p.A., ref X0043) was used to prepare the stools for the Liaison calprotectin immunoassay. The sampling wand of the device is dipped into the stool sample multiple times (3–5 times), tightly screwed onto the device and 6 ​mL of extraction buffer is added. The samples are then homogenized on a multi-tube vortex mixer for 30 ​min. After homogenization, the stool extract is ready for analysis on the LIAISON® XL analyser without any sample transfer. In this study the stability of the extract was tested according to the manufacturer’s instructions (data not shown). The EliaA Stool Extraction Kit 2 was used according to the manufacturer’s instruction to prepare the stools for the EliA calprotectin immunoassay. The sampling rod was dipped into the stool multiple times (3–5) and tightly screwed onto the device with extraction buffer. The stool sample was homogenized using vortex. Afterwards, samples were incubated at room temperature for 10 ​min. The homogenate was transferred to an Eppendorf tube and centrifuged for 5 ​min at 3000 ​g. The supernatant was then transferred to a fresh tube and ready for testing.

### 2.3 Fecal calprotectin assays

The fecal calprotectin immunoassay was conducted in the LIAISON® XL analyser. We used the protocol according to the manufacturer (DiaSorin S.p.A., ref 318960). This analyser uses a CLIA for the quantitative determination of calprotectin in human stool specimens. Calibrators, controls and samples were analysed according to the manufacturer’s instructions. The amount of light measured is directly proportional to the amount of calprotectin presence. For this study, calprotectin measurements were performed 3 times a week in a batch-like procedure using the same lot of reagent throughout the study. The calprotectin concentration in the sample was determined on the standard curve, and expressed in micrograms of calprotectin per gram of feces (μgC/g feces, which is equal to mg C/kg feces). If the sample value exceeded 800 ​mg/kg, the sample was automatically diluted 1:10 with Diluent and re-measured. Due to the optional dilution step, the LIAISON® Calprotectin assay DiaSorin can measure up to 8000 ​mg calprotectin per kilogram of feces. If no additional dilution of the samples is needed, the run time for this assay is of 35 ​min. Another 2 ​min per sample if dilution is required (*de novo* measurement).

Measurements obtained from the DiaSorin’s LIAISON® XL analyser using the LIAISON® Calprotectin test were compared to the TFS EliA calprotectin 2 immunoassay (EIA) conducted on the ImmunoCAP250 analyser. The amount of fluorescence measured after the enzymatic reaction is directly proportional to the amount of calprotectin in the stool sample. The run time of this assay is 1 ​h and 45 ​min.

### 2.4 Analytical assay validation

The linearity of the measurements was assessed by combining two stools samples of respectively 716 ​mg/kg and 7 ​mg/kg in the following manner (high:low): 4:0, 3:1, 2:2, 1:3, 0:4. To assess a possible interference of the diluent buffer a stool sample of 835 ​mg/kg was measured after being diluted in 4 steps in the following manner (sample:diluent): 4:0, 2:2, 1:4, 1:9, 1:19. The measured results were compared to the calculated concentrations. The intra and inter-run variability were evaluated following CLSI guideline (EP5-A2) using two calibrators provided by DiaSorin (Control 1:42.5–75.6 ​mg/kg; Control 2:200–356 ​mg/kg). The claimed repeatability and within-laboratory precision were established *a priori* by the manufacturer. The reproducibility in the lower measuring range (i.e. around 5 ​mg/kg since lower concentrations are reported <5 ​mg/kg) was measured by calculating the variation coefficients of 3 stool samples in the lower measuring range after each sample was measured 9–10 times.

To asses a possible interference of blood in the stool, several experiments were carried out. Initial testing was done on a stool sample that tested negative for occult blood. Either 150 ​μl of blood (from a patient’s venous sample) or 150 ​μl of blood from isolated erythrocytes provided by our Transfusion unit within the laboratory were added to the stool sample before extracts were obtained. Calprotectin concentrations were measured using the calprotectin immunoassay from DiaSorin before and after blood was added.

Further testing was conducted using the TFS′ EliA calprotectin kit. The first test was designed to assess whether the interference was stool-dependent. A set of 10 different stool samples were measured before and after the same blood sample (patient’s or erythrocyte package unit) was added with a fixed Hb of 10 ​mmol/l measured in the blood sample (same [Hb], different stools). For this purpose 60 ​g (40 ​g of stool and 10 ​ml of NaCl buffer) of 10 different stool samples were divided into 3 portions. The first portion was used for an occult blood test (which was always negative) and for the baseline calprotectin measurement. In the other 2 portions, calprotectin was measured by adding 150 ​μl of blood (from a patient’s venous sample) or 150 ​μl of blood and from isolated erythrocytes to the stool portion. The second test was designed to assess whether the interference was Hb-dependent. In this case, one stool sample was divided into 10 parts and each was measured before and after adding a blood sample (same stool, different [Hb] ranging from 6,8-9,4 ​mmol/L as measured in the blood samples). For this purpose, 60 ​g (40 ​g of stool and 10 ​ml of NaCl buffer) were divided into 21 portions: 1 as control, 10 on which venous blood was added and 10 on which isolated erythrocytes were added.

For analytical method comparison, the 303 stool samples used for the clinical validation were previously measured on the ImmunoCAP250 [19] using the TFS’ EliA calprotectin kit. A set of calprotectin standards were prepared using a recombinant human calprotectin antigen provided by the manufacturer. They were diluted in deionized water to the equivalent of a series of stools after extraction ranging from 20 to 600 ​mg/kg to study possible matrix differences between the assays. Passing&Bablok and Bland-Altman analysis was conducted using Analyse-it.

### 2.5 Clinical validation

The diagnostic performance of the calprotectin immunoassay from DiaSorin was assessed in IBD patients and compared to the EliA calprotectin 2 immunoassay from TFS. 303 samples were measured from 5 different groups (see Table 1) including UC, CD, IBS, other GI diseases and controls. An additional set of 9 samples from different IBD patients (i.e. UC and CD), were measured upon diagnosis and after treatment was started (follow-up study). Based on the results, specificity, sensitivity, positive predictive value (PPV) and negative predictive value (NPV) were calculated. The clinical validation was conducted by one-way ANOVA using the Graphpad Prism 5 package (La Jolla, CA, USA).

## 3 Results

### 3.1 Analytical performance

The overall analytical performance of the LIAISON® Calprotectin immunoassay from DiaSorin was outstanding. This new assay opens the possibilities of standardization due to the use of human recombinant standards for the first time. This is necessary when taking into consideration the already reported differences between available methods [18].

### 3.2 Linearity

**Fig. 1:**
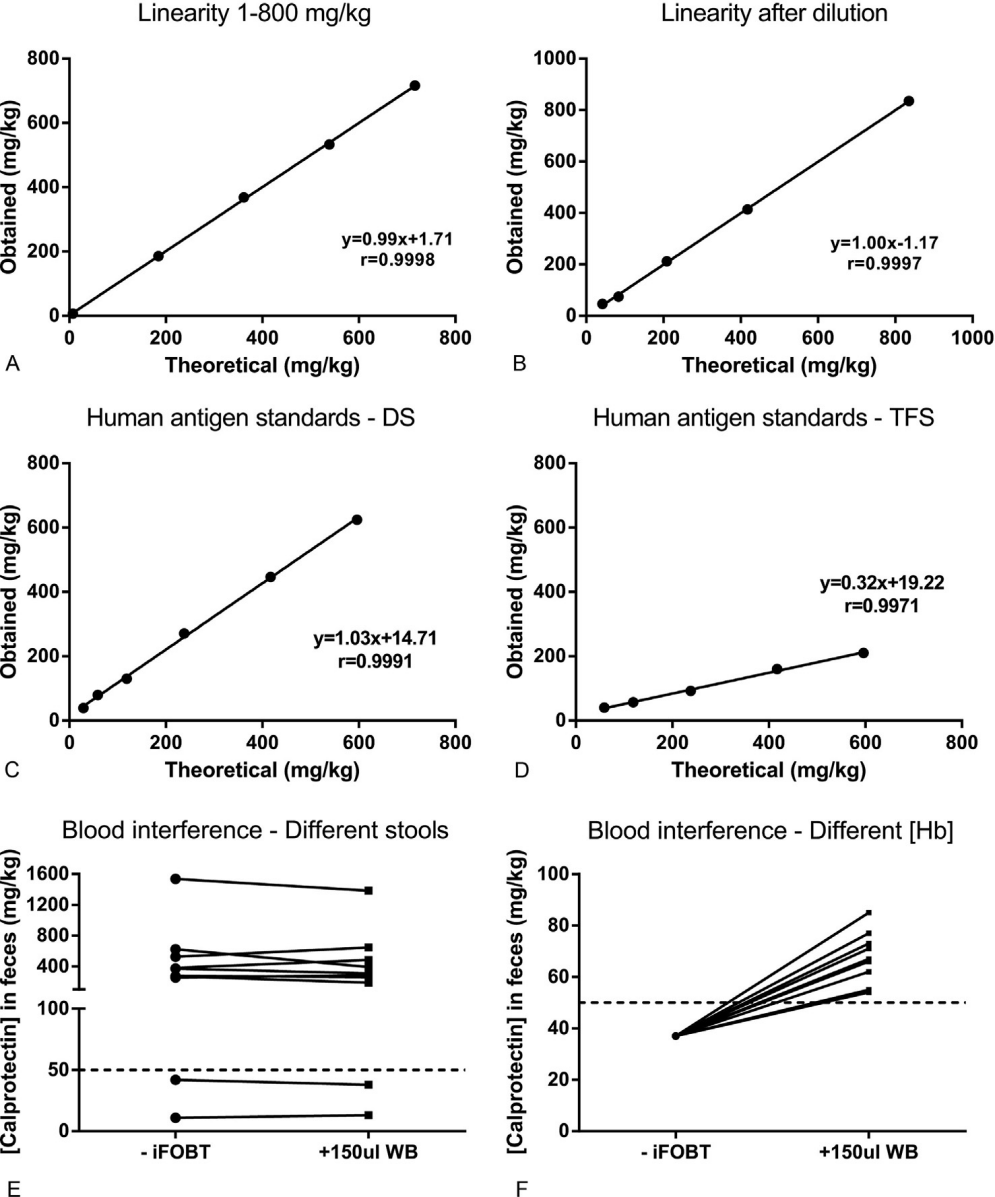
**A, B.** Linearity measurements over the concentration range of 5 to 800 ​mg/kg **(A)** and after dilution using diluent buffer **(B)**. Linearity curves were obtained plotting measured calprotectin concentrations on the y-axis vs. the theoretical values on the x-axis. **C, D.** Set of standards containing a recombinant human calprotectin antigen measured with the DiaSorin assay on the LIAISON® XL **(C)** and with the Thermo Fisher Scientific assay on ImmunoCAP250 **(D)**. **E, F.** Blood interference in the calprotectin measurement with the Thermo Fischer Scientific assay. **E.** Calprotectin concentration measurements before (-FOBT) and after (+150 ​μl WB) the adding of whole blood from the same patient to 10 different stool samples. **B.** Calprotectin measurements from the same stool sample before (-iFOBT) and after (+150 ​μl WB) adding whole blood from 10 different patients.

The linearity of the measurement resulted in the linear regression equation: y ​= ​0.99x+1.71 with a correlation of 0.9998 (p ​< ​0.0001, Fig. 1a) after combining two stools samples of respectively 716 ​mg/kg and 7 ​mg/kg in 4 different ways. After dilution in 4 steps (from undiluted to up to 1:19 dilution) of a stool sample of 835 ​mg/kg the linear regression equation: y ​= ​1.00x-1.17 with a correlation of 0.9997 (p ​< ​0.0001, Fig. 1b) was obtained. This shows that an increase or decrease of disease activity within a patient can be monitored. For more information see the analysis on follow-up patients in the clinical validation section. As previously stated, a set of human antigen standards were diluted in deionized water and measured with both immunoassays, resulting in the linear regression equation: y ​= ​1.03x+14.71 with a coefficient of 0.9991 (p ​< ​0.0001) (Fig. 1c) for the DiaSorin assay and y ​= ​0.32x+19.22 with a correlation of 0.9971 (p ​< ​0.0001) (Fig. 1d) for the TFS assay. These results’ deviation from the estimated range, suggest that an unknown factor between assays is present.

### 3.3 Precision

The intra-run CV (%) was well within the established goals, for control 1 (42–75 ​mg/kg) was 2,7% (goal <6,0%) and for control 2 (200–356 ​mg/kg) was 3,6% (goal <6,7%). The inter-run CV for control 1 was 5,1% (goal of <6,3%) while the inter-run for control 2 was 4,7% (goal of <4,2%) and therefore did not meet the pre-established criteria. However, it was well within the limits reported in previous studies [15,18]. Further, we calculated the coefficient of variation for 3 stool samples within the lower region of the measuring range resulting in CV of 6% (sample of 8 ​mg/kg), 3% (sample of 11 ​mg/kg) and 6% (sample of 12 ​mg/kg). All CVs were well under the established 20% cut-off.

### 3.4 Blood interference

Within the lab, occult blood can be measured in fecal samples but it is not common practice in IBD patient’s samples, even though it is a common symptom among these patients. Before adding blood to a single stool sample, calprotectin concentration measured with DiaSorin’s immunoassay was 169 ​mg/kg. After adding blood (either a randomly chosen patient’s whole blood or isolated erythrocytes), calprotectin concentrations were 137 ​mg/kg (reduction of 19%, patient’s blood), and 141 ​mg/kg (reduction of 17%, isolated erythrocytes) respectively. We studied the blood interference conducting additional experiments using the TFS′ immunoassay in which blood interference was also observed (as for DiaSorin’s immunoassay). The first experiments using a single patient’s whole blood (same Hb, different stool Fig. 1e) in 10 different stool samples showed different effects (reduction or increase) per stool, independent of the calprotectin concentration. The second set of experiments using the same stool sample split in 10 aliquots each with a different patient’s blood sample (same stool, different [Hb] Fig. 1f) showed a false higher calprotectin concentration for all different [Hb]. There was no apparent relation between increase in calprotectin concentration and Hb concentration. The same set of experiments was repeated with isolated erythrocytes from a transfusion unit yielding similar results (data not shown). These experiments show that the effect of blood, when measuring calprotectin, is feces-dependent and not blood-dependent. The effect itself cannot be predicted beforehand and can lead to false results (changes from −40% up to +130% in comparison with original measurement).

#### 3.4.1 Method comparison

**Fig. 2:**
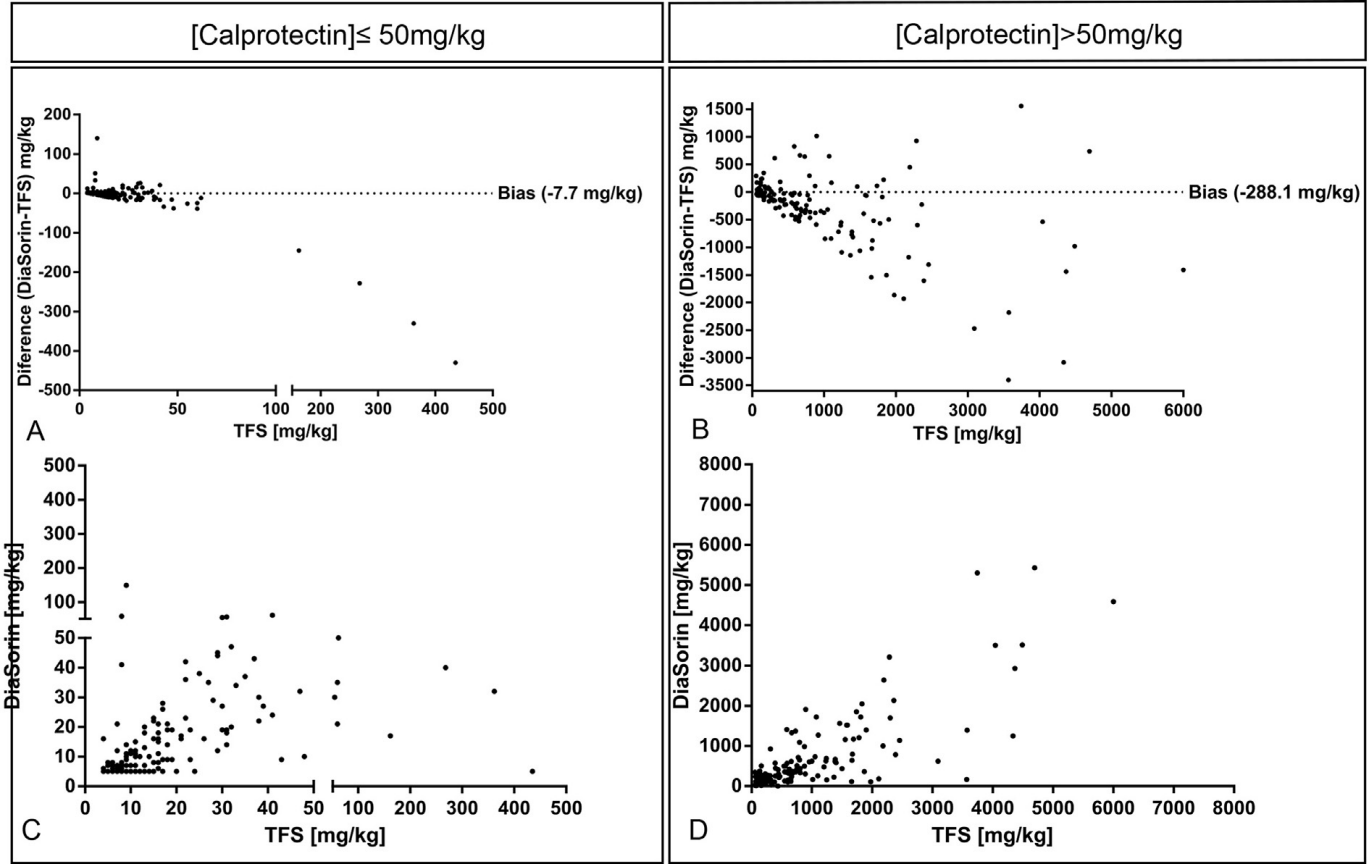
Bland-Altman plots of method comparison (n ​= ​303) for DiaSorin calprotectin assay and established Thermo Fisher Scientific (TFS) assay. Absolute (A ​+ ​B) and percentage (C ​+ ​D) Bland-Altman plots, respectively. FC, fecal calprotectin.

Calprotectin concentration was measured in all 303 samples using both immunoassays, which were compared by Passing and Bablok regression analysis. The method comparison was divided into two groups: one for the samples under the diagnostic cut-off value of ≤50 ​mg/kg and the other for samples >50 ​mg/kg. The resulting regression equations were y ​= ​0.78x+ 1.09 and y ​= ​0.70x-15.70 respectively. Bland-Altman analysis outlined a significant bias for the DiaSorin calprotectin assay (Fig. 2 and Table 2). A qualitative comparison between assays was conducted to assess the validity of DiaSorin’s immunoassay. From the 303 tested samples, 158 were found negative (i.e. ≤50 ​mg/kg) 145 were found positive (i.e. >50 ​mg/kg) using DiaSorin’s assay (Table 3). Using TFS′ assay, of the 303 samples, 151 were found negative and 152 positive (Table 4). Four samples resulted positive using the DiaSorin assay while they resulted negative using the TFS′ assay. Another 11 samples resulted negative using the DiaSorin assay and positive with the TFS assay (Table 5).

**Table 2:** Method comparison between Liaison Calprotectin on DiaSorin’s Liaison XL and TFS EliA Calprotectin on ImmunoCAP250.

	[Calprotectin] ≤50 ​mg/kg	Calprotectin >50 ​mg/kg	All data
**Passing-Bablok regression**			
Slope (95% CI)	0.78 (0.63–0.96)	−15.70 (−57.41 to 30.36)	0.69 (0.64–0.76)
Intercept (95% CI)	1.09 (−0.73 to 2.32)	0.70 (0.59–0.80)	1.57 (0.46–2.45)
**Bland-Altman analysis**			
Bias (95% CI) mg/kg	−7.7 (15.7–0.2)	−288.1 (−399.9 to −176.2)	−123.4 (−197.0–49.8)
Bias (95% CI) %	−15.5 (−24.5 to −0.6)	−37.9 (−49.1 to −26.8)	−24.6 (−31.8 to −17.4)

**Table 3:** Concordance table for the Liaison® Calprotectin assay for the stool samples measured in this study.

	Disease	No disease	Total	
**Positive** ​> ​**50 ​mg/kg [calprotectin]**	121	24	145	**PPV:83%**
**Negative** ​< ​**50 ​mg/kg [calprotectin]**	6	152	158	**NPV: 96%**
**Total**	127	176		
	**Sensitivity: 95%**	**Specificity:86%**

**Table 4:** Concordance table for the ELiA Calprotectin assay for the stool samples measured in this study.

	Disease	No disease	Total	
**Positive** ​> ​**50 ​mg/kg [calprotectin]**	124	28	152	**PPV:82%**
**Negative** ​< ​**50 ​mg/kg [calprotectin]**	3	148	151	**NPV: 98%**
**Total**	127	176		
	**Sensitivity: 98%**	**Specificity:84%**

**Table 5:** Overview of calprotectin conclusion discrepancies between the Liaison® calprotectin (DiaSorin (DS)) and the EliA calprotectin (Thermo Fisher Scientific (TFS)) assays. ∗: Crohn’s disease patients or ulcerative colitis patients showing a false negative result for calprotectin concentrations using the Liaison® calprotectin assay.

	Gender	Age	TFS (mg/kg)	DS (mg/kg)	Diagnosis
**Positive Liaison® (DS), Negative EliA (TFS)**	Female	59	30	55	Crohn-like diarrhoea with no clear focus. Endoscopy revealed no IBD and after the patient adjusted her diet, the complaints disappeared.
	Female	25	31	58	IBS.
	Female	47	8	60	Altered defecation patterns with a family history of colon carcinoma.
	Male	26	9	149	Obstipation problems.
**Negative Liaison®, Positive EliA (TFS)**	Female	43	268	40	Pain in the lower left abdomen. IBS.
	Female	45	60	35	Chronic abdominal complaints, low ferritin and vitamin D levels, possible thyroid problem. IBS.
	Female	53	60	21	IBS-related symptoms in well controlled Crohn’s disease patient.
	Male	33	62	50	Pancreas failure.
	Male	45	362	32	Gastroenteritis.
	Male	50	55	29	Diarrhoea e.c.i.
	Male∗	67	435	<5	Crohn’s disease and Turberculose suspicion
	Female∗	72	162	17	Diarrhoea and abdominal pain, relapse in Crohn’s disease patient.
	Male∗	51	328	32	Ulcerative colitis patient with symptoms that were somewhat receding.
	Male∗	65	93	28	Mildly active pancolitis patient.
	Female∗	63	95	45	Pancolitis patient.

From the collected data a clear difference between assays is observed. However, the difference does not seem proportional, in other words, a specific factor cannot be determined. To assess whether this difference may be explainable by the use of different extraction kits (including buffers), a patient sample was measured first with the DiaSorin assay in the LIAISON® XL and immediately after on the ImmunoCAP250 (with the DiaSorin extraction buffer). The calprotectin concentration measured on the LIAISON® XL was 305 ​mg/kg and on the ImmunoCAP250, 102 ​mg/kg. This result suggests that the differences observed between the assays cannot be explained by the different extraction kits used.

### 3.5 Clinical performance

**Fig. 3:**
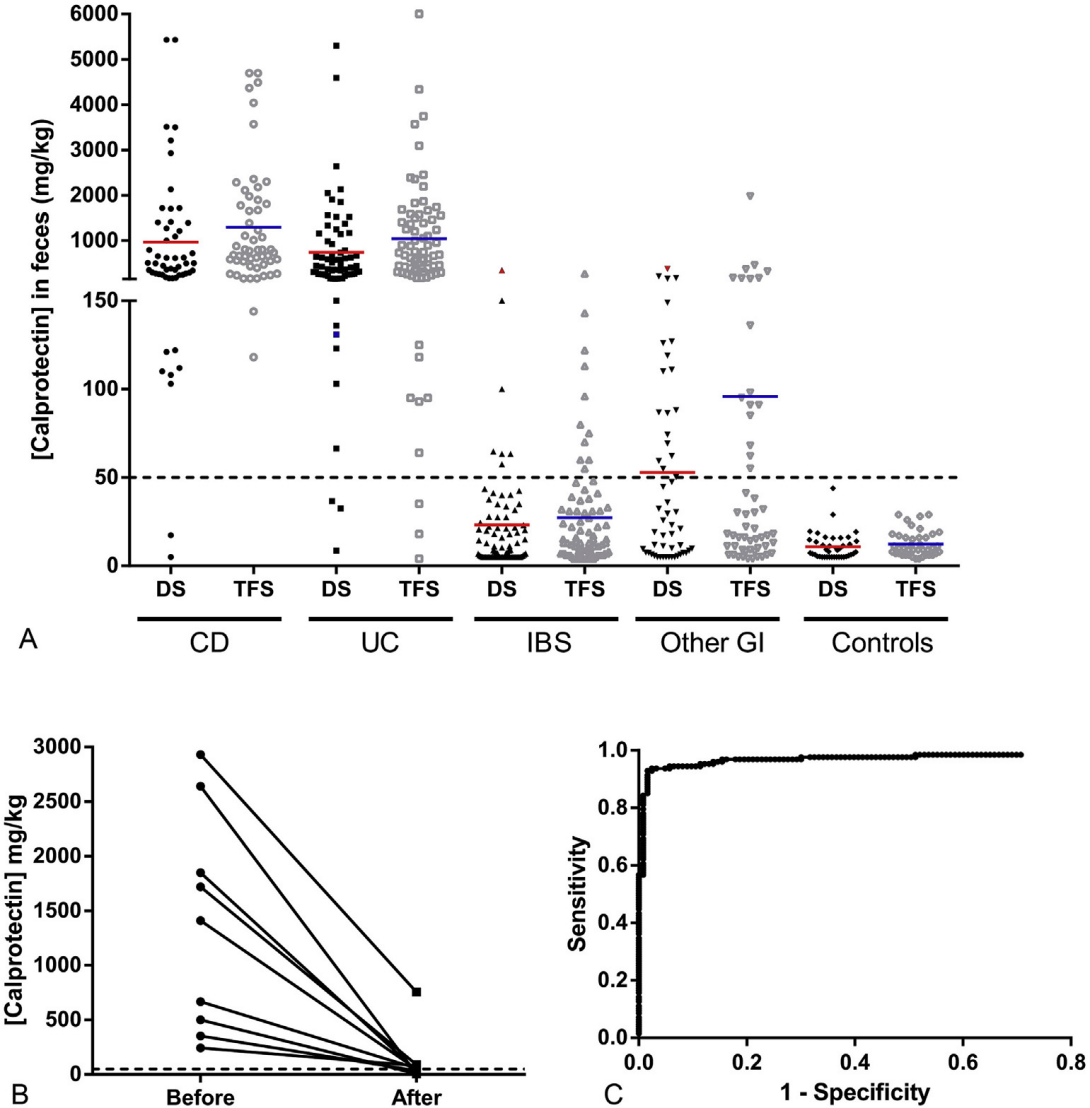
Clinical validation of the DiaSorin calprotectin assay on the LIAISON®XL. Calprotectin concentrations measured for the different patient groups with DiaSorin’s immunoassay (DS) and Thermo Fisher Scientific’s (TFS) immunoassay **(A)**. Follow-up results before and after treatment was started in a subset of patients measured with DiaSorin’s immunoassay **(B)**. CD: Crohn’s disease; UC: ulcerative colitis; IBS: irritable bowel syndrome; Other GI: other gasto-intestinal disease.

The results of calprotectin measurements for each of the 303 patient stool samples (divided into 5 groups) measured with both immunoassays (DiaSorin and TFS) can be seen in Fig. 3A. When comparing all the groups measured with DiaSorin’s immunoassay, our results show a significant difference in calprotectin concentrations between IBD patients (i.e. CD and UC), non IBD patients (i.e. IBS and other GI diseases) and controls. The same can be said for the measurements obtained with TFS′ immunoassay. From all diagnosed IBD patients, a total of 5 showed calprotectin concentrations below the cut-off value of 50 ​mg/kg with DiaSorin’s immunoassay. These patients are described in Table 5 because the concentrations measured between the immunoassays yielded different conclusions (>or ≤50 ​mg/kg cut-off value). From the 303 samples measured with DiaSorin’s immunoassay, a sensitivity of 95% and specificity of 86% can be calculated (Table 3). At the 50 ​mg/kg cut-off value, the negative predictive value (NPV) of DiaSorin for detecting IBD was 96% and the positive predictive value (PPV) was 83%. The results obtained with the immunoassay from TFS yielded a higher sensitivity of 98% and a similar specificity of 84% (Table 4). At the same cut-off value of 50 ​mg/kg, the NPV of TFS for detecting IBD was 98% and the PPV was 82%.

For the follow-up study, a group of 9 patients newly diagnosed with either UC (4/9) or CD (5/9) were measured upon diagnosis and between 2 to 3 months after treatment had started. These patients were treated with different agents (azathioprine, mercaptopurine, infliximab, adalimumab or budesonide) depending on the diagnosis and extent of inflammation. The results are shown in Fig. 3B.

## 4 Discussion

In this study we evaluated the analytical and diagnostic performance of the fecal calprotectin immunoassay developed by DiaSorin for the LIAISON® XL. This new immunoassay presents a handful of advantages in measuring calprotectin in the laboratory settings. First, using DiaSorin’s calprotectin stool extraction device is and easy 2-step protocol which allows for big batches to be prepared for analysis at once, providing the laboratory with the opportunity to shorten the turn around time of the results provided to the physicians. Using EliA’s stool extraction kit involves multiple manual steps. Although each step individually is shorter than DiaSorin’s 2-step protocol, in our hands, overall preparation time was more labour intensive, resulting in a smaller batch size in the same amount of time. The average batch size using DIaSorin’s method is of 60 samples as opposed to 30 samples using TFS’s method. The preparation time for both methods is the same, around 1,5 to 2 ​h. The incidence of patients suffering from IBD is increasing [1]. Since the value of calprotectin measurements for diagnosis and monitoring of IBD has been shown [9,20], the demand for calprotectin testing has increased as well. A quick and easy use of the extraction device enables an increase of the samples processed by the laboratory. Second, DiaSorin’s calprotectin assay shows favourable analytical performance. The measurement shows good linearity before and after dilution, expanding the measuring range slightly, providing more information on follow-up of patients with high calprotectin concentrations. The analytical precision is excellent showing an inter-run CV for control material (low and high concentrations) as well as for stool samples in the low range (6–12 ​mg/kg) of around 6%. A previous study reported for the EliA Calprotectin immunoassay on ImmunoCAP250 an inter-run CV% of 9.7% in stool sample with calprotectin concentration of 137 ​mg/kg. [17] Another study reported intra-run and total imprecision (% CV) for both control materials as well as stool samples of between 5.7% up to 9.1% for the EliA Calprotectine 2 immunoassay on the ImmunoCAP250 and 2.1% up to 7.2% for the DiaSorin immunoassay on the Liaison. [18].

However, some crucial aspects when measuring calprotectin concentration were identified in this study. First, the extraction step (pre-analytical step) gave the highest variability to the calprotectin measurement. This is a common problem for all available extraction devices.

As such, the imprecision of this step had to be evaluated. When conducted by the same technician on normal stools, our results show VCs from 5% to 18% (data not shown). Previous studies have already shown that commercial extraction devices undersample when compared to the manual extraction method weighing a specific amount of stool (golden standard, stool weight method) [15]. The difference becomes more evident when dealing with watery stools. These were not included in the study because, at the time, the extraction device could not be used for watery stools according to the manufacturer. However, given that IBD patients can often present watery, bloody stool during exacerbation, the manufacturer presented us a way to analyse these samples using the stool weight method. When using this assay in the real-life setting of the lab, it is recommended to limit the amount of technicians involved in the extraction process thus attempting to limit variation between samples. Additional pre-analytical variability can be introduced as has recently been reported [21] including storage temperature and time. When measuring calprotectin in the laboratory it is important to standardize the process to minimize variability between measurements for the same patient.

Another important key finding in this study is that the presence of blood in the stool can interfere with the measurement of calprotectin. Bleeding is quiet common in IBD patients, especially when disease activity is high. Up to date, the possible interference of blood when measuring fecal calprotectin has not been reported. A previous study shows no interference of blood in the measurement using the Gentian Calprotectin immunoassay [22], however in our hands, this interference is observed for both immunoassays (DiaSorin and TFS). It is feces-dependent, which suggests that other factors are responsible for the variation, like for example diet. This poses an interesting point since at least in our hospital fecal occult blood tests are not conducted prior to calprotectin measurement. On the other hand, the effect of blood in the analysed samples did not show an effect on the clinical outcome. A further understanding of this interference is required. When analysing the possible interference of blood in the measurement, variation due to extraction should be taken into account. In our hands, a variation of up to 18% was see when different extractions were conducted on the same stool sample, however, it cannot account for the up to +130% variation seen with blood interference.

The comparison between methods (DiaSorin and TFS) yielded some important findings. The difference between assays does not seem proportional. Previous studies where the DiaSorin’s assay is compared to other assays, showed a proportional difference with the fCAL™ turbo (Bühlmann Laboratories AG) on the Cobas C501 (Roche) or the Bühlmann ELISA method [16] and a significant difference with the point of care test Quantum Blue® from Bühlman-Alere [23]. A matrix effect derived from the extraction step could account for the differences in measurements. However, even after limiting any possible matrix effect, the difference between both assays remained. The lack of international standardization for fecal calprotectin measurement does not help in resolving measurement differences. The commercial assays have antibodies targeting different calprotectin epitopes that could account for the differences. Another possibility lies on differences introduced during the extraction step either by the lab technician or by the used extraction device. Using the same extraction kit (and buffer) yielded different results between the two analysis platforms (Liaison XL and ImmunoCAP250), suggesting that the extraction buffer does not play a big role in the differences observed. The measurement of human standards dissolved in deionized water yielded differences between platforms that, at this time, could not be accounted for. Taken together, this proves that several factors involved in calprotectin’s immunoassays account for the method variability, making the need of a Joint Committer for Traceability in Laboratory medicine (JCTML) standard for calprotectin a high priority. Even though previous studies have shown the differences between the extractions kits and the weight method, overall, the clinical interpretation of the results does not change [[16], [17], [18],^22^]. In the study by Oyaert and colleagues, to prevent pre-analytical variation, all samples were weighted and extracted using the same kit [18]. Results still showed clear differences between methods, however, qualitative correlation was good. This means that quantitative results cannot be compared between methods raising an important issue, at least in The Netherlands. Currently, a total of 5 different assays are used, without counting the different extraction kits. Thus, when a patient moves between hospitals or laboratories it is important to note that, although the qualitative result should be comparable, the quantitative result obtained in the current hospital cannot be compared with one obtained elsewhere.

Having said this, the diagnostic accuracy of calprotectin measured by DiaSorin’s immunoassay is high. Different studies have reported a sensitivity and specificity of 89% and 81% [18] (n ​= ​1267) and 83% and 84% [24] respectively for calprotectin in the diagnosis of IBD. Fecal extracts in these studies were obtained following the ‘manual’ approach as previously described [25]. These values have increased in more recent studies up to sensitivities of >93% and specificities of 96% [9,16]. Our findings concur with the existing data using different extraction kits and immunoassays yielding a sensitivity of 95% and specificity of 86% at the 50 ​mg/kg cut-off point. Despite the apparent method differences, the clinical interpretation of the measurements obtained with DiaSorin’s immunoassay is accurate. Distinction between IBD and non-IBD patients can be made. It is interesting to note that the TFS assay measured considerably higher levels of calprotectin in the group of other gastrointestinal diseases than DiaSorin’s assay. The underlying explanation still needs to be elucidated. Also, patient follow-up showed that DiaSorin’s immunoassay can provide accurate therapy-related results.

## 5 Conclusions

DiaSorin’s LIAISONXL® Calprotectin immunoassay showed good analytical and clinical performance being suitable for detection of IBD and follow-up during therapy. The assay has a shorter extraction time and the time of measuring is much shorter making it very suitable for laboratories with high throughput of samples.

## CRediT authorship contribution statement

**R. Vicente-Steijn:** Conceptualization, Methodology, Validation, Formal analysis, Writing - original draft, Project administration. **J.M. Jansen:** Validation, Supervision. **R. Bisheshar:** Validation, Investigation. **I.-A. Haagen:** Conceptualization, Writing - review & editing, Supervision, Project administration.

## Declaration of competing interest

None.
